# Impact of aging on transition of acute kidney injury to chronic kidney disease

**DOI:** 10.1038/s41598-019-54585-1

**Published:** 2019-12-05

**Authors:** Myung-Gyu Kim, Jihyun Yang, Yoon Sook Ko, Hee Young Lee, Se Won Oh, Won Yong Cho, Sang-Kyung Jo

**Affiliations:** Division of Nephrology, Department of Internal Medicine, Korea University Anam Hospital, Seoul, Korea

**Keywords:** Acute kidney injury, End-stage renal disease

## Abstract

Acute kidney injury (AKI) increases the risk of end stage renal disease among the elderly, but the precise underlying mechanism is unknown. We investigated the effects of aging on AKI-to-chronic kidney disease (CKD) transition, focusing on renal inflammation. Aged and young C57BL/6 mice were subjected to bilateral ischemia-reperfusion injury (IRI). Baseline proinflammatory cytokine levels of kidneys were elevated in aged mice. After IRI, aged mice also showed persistent M1 dominant inflammation, with increased proinflammatory cytokines during the recovery phase. Persistent M1 inflammation was associated with blunted activation of CSF-1/IRF4 signal for M1/M2 polarization, but *in vitro* macrophage polarization with cytokine stimulation was not different between young and aged mononuclear cells. The tubular expressions of cell cycle arrest markers increased in aged mice during recovery phase, and *in vitro* transwell experiments showed that mononuclear cells or M1 macrophages co-cultured with arrested proximal tubular cells at G1 phase significantly impaired M2 polarization, suggesting that prolonged G1 arrest might be involved in persistent M1 inflammation in aged mice. Finally, M1 dominant inflammation in aged mice resulted in fibrosis progression. Our data show that impaired M2 polarization partially driven by senescent tubule cells with cell-cycle arrest may lead to an accelerated progression to CKD in the elderly.

## Introduction

As the population ages, the incidence and prevalence of noncommunicable diseases such as diabetes, hypertension, chronic kidney disease (CKD), and cancer are growing rapidly. According to national registry data, the percentage of elderly (≥65 yrs) end stage renal disease (ESRD) population has shown a steady increase worldwide^1–4^.

Several epidemiological studies have reported a high prevalence of acute kidney injury (AKI) requiring dialysis in elderly patients, suggesting that aging might increase the severity of AKI^5–8^. Disturbance of autoregulation/hemodynamics or structural changes in aged kidneys^9,10^ seem to be contributing factors. In addition, AKI in the elderly has been shown to increase the risk of progression to CKD/ESRD, even after adjusting for important covariates^5,11,12^. However, this relationship mostly comes from epidemiological studies of elderly patients with comorbid conditions, and the mechanisms responsible for possible impaired recovery after AKI in the elderly have not been thoroughly investigated.

The immune system undergoes a dynamic change with aging, characterized by chronic low-grade inflammation, called inflammaging^13^. Reduced numbers of naïve T and B cells and increased levels of proinflammatory cytokines in cells of myeloid origin have been reported. In addition to intrinsic immunosenescence, cellular senescence or altered phenotype of nonimmune cells in tissue microenvironments might also have an effect on the phenotype of senescent immune cells.

Macrophages are cells of substantial plasticity and have been shown to play an important role in both injury and recovery in animal models of AKI^14–17^. While classically activated M1 macrophages contribute to initial injury, conversion to M2 anti-inflammatory macrophages during the recovery phase is critical in resolving inflammation and restoring normal architecture and function. However, most studies have been performed in young mice, and data showing the effect of aging on injury/repair processes or macrophages phenotypes in AKI are lacking.

In this study, we investigated the effects of aging on the injury/repair process of AKI in a mouse model of ischemia reperfusion injury (IRI), with a focus on altered macrophage phenotype in the context of kidney microenvironment. We found that impaired macrophage M2 polarization driven by adjacent tubule cells with a cell cycle arrest phenotype is partially responsible for impaired recovery from AKI in aged mice.

## Results

### Effect of aging on acute kidney injury severity

**Figure 1:**
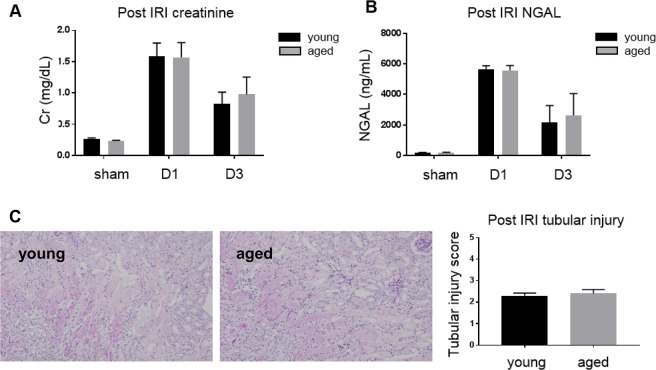
Renal functional deterioration and histologic damage following ischemia-reperfusion injury (IRI) of young and aged mice. There is no difference in (**A**) serum creatinine, (**B**) NGAL, and (**C**) tubular injury scores on day 1 and 3 after IRI between young and aged mice. Magnification: ×100, n = 4–6 per group.

Functional deterioration and histologic damage were compared after IRI. Serum creatinine, neutrophil gelatinase-associated lipocalin (NGAL), and tubular injury scores on days 1 and 3 were not significantly different between aged and young mice (Fig. 1).

### Effect of aging on kidney inflammation in ischemia-reperfusion injury

**Figure 2:**
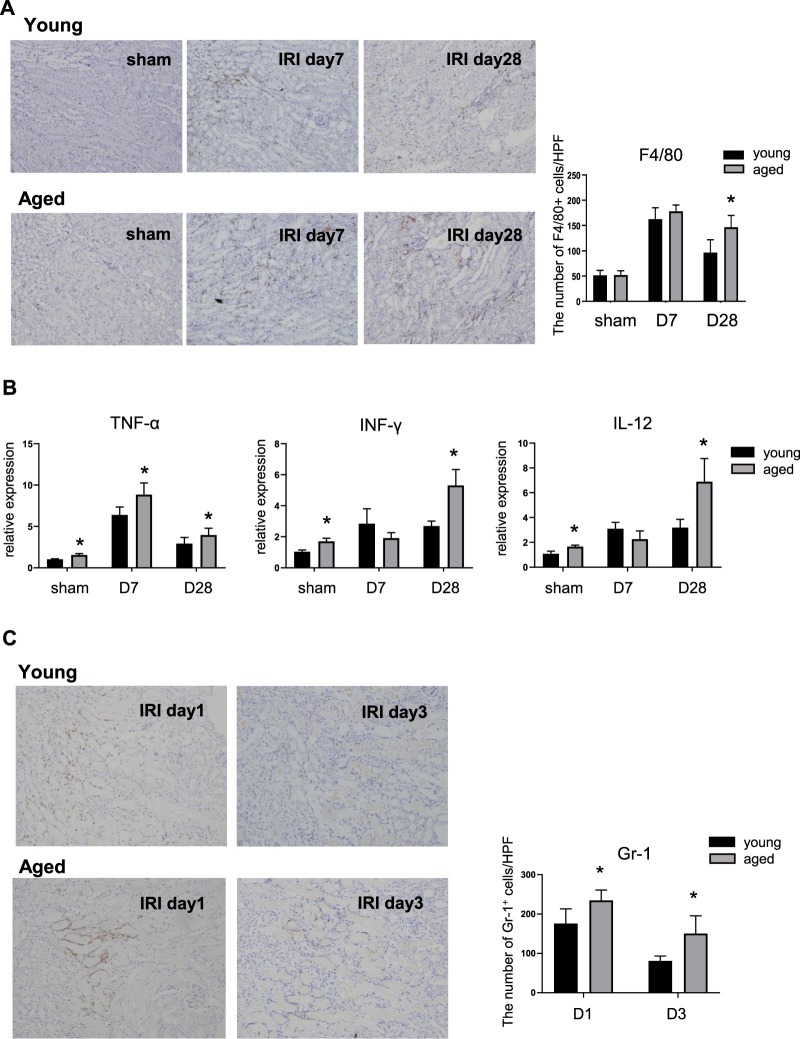
Renal inflammation during recovery phase of young and aged mice. (**A**) In immunohistochemical staining, infiltration of F4/80^+^ macrophages increased during recovery phase in both young and aged mice and remained for a significantly longer duration in aged mice than in young mice, (**B**) Prior to IRI, the renal expression of TNF-α, IFN-γ, and IL-12 was higher in aged mice than in young mice, suggesting the presence of low-grade inflammation. After IRI, they were increased more in aged mice than in young mice and the difference was greater on day 28, (**C**) The number of neutrophils was also significantly higher in aged kidneys. Magnification: ×100, *p < 0.05 compared to young mice, n = 4–6 per group.

Despite comparable numbers of F4/80 + resident macrophages, baseline levels of kidney TNF-α, IFN-γ, and IL-12 mRNA were significantly elevated in aged mice (sham) compared to young mice, suggesting the presence of chronic low-grade inflammation. On day 1 after IRI, the number of macrophages increased significantly in both young and aged mice. However, mRNA levels of TNF-α, IFN-γ and IL-12 in aged mice were significantly higher than those of young mice. Throughout the recovery phase, young mice showed decreased number of macrophages and inflammatory cytokines; these changes were markedly blunted in aged mice, with a significantly higher number of macrophages and tissue levels of proinflammatory cytokines on day 28 of IRI (Fig. 2A,B). The number of neutrophils was also significantly higher in aged kidneys, showing the lack of resolution of neutrophilic inflammation (Fig. 2C).

### Impaired M1-M2 polarization during the recovery phase of ischemia-reperfusion injury in aged mice

**Figure 3:**
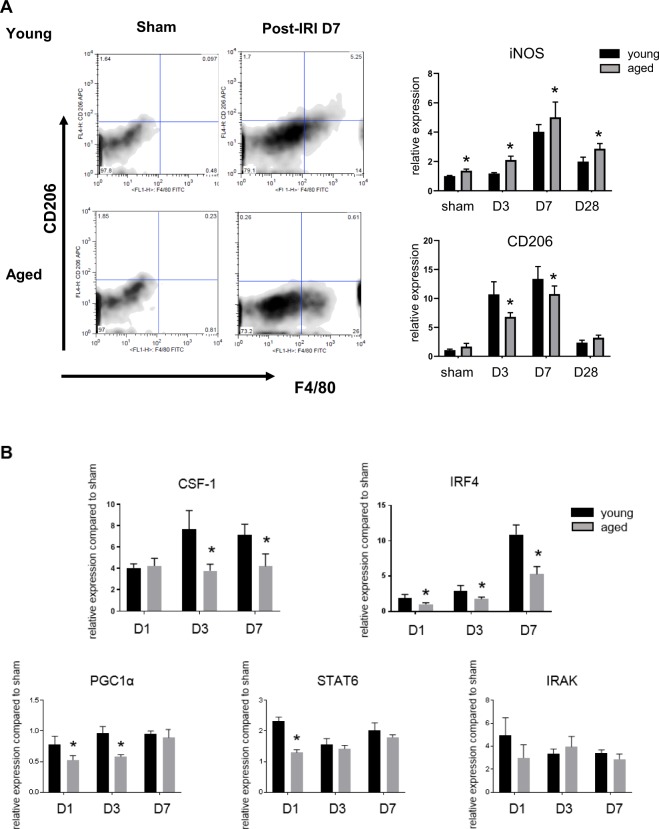
Impaired M1-M2 polarization during recovery phase in aged mice. (**A**) Renal macrophages of aged mice were skewed from the “F4/80 + CD206 + M2” to “F4/80 + CD206- M1” compared to those in young mice during recovery phase, (**B**) The increase in mRNA expressions of colony stimulating factor-1 (CSF-1), interferon regulatory factor-4 (IRF4), peroxisome proliferator-activated receptor gamma coactivator 1-alpha (PGC-1α) were blunted in aged mice, but mRNA expressions of STAT6 and IL-1 receptor-associated kinase-M (IRAK-M) were not. *p < 0.05 compared to young mice, n = 4–6 per group.

Flow cytometry of kidney mononuclear cells showed that the number of kidney F4/80 + CD206 + M2 macrophages on day 7 after IRI was significantly lower in aged kidneys with increased iNOS/decreased arginase expression (Fig. 3A). In kidney, M2 macrophages can be derived from *in situ* proliferation of resident macrophages, differentiation from infiltrating monocytes or phenotype switch from M1^18^. And disturbances in these processes can interfere with the growth of M2 populations during recovery phase of IRI. Although it is difficult to differentiate the contribution of each process to M1/M2 imbalance in aged mice, we were interested in whether there is an impairment of M2 polarization during recovery phase, because recent studies have reported that M2 macrophages in the IRI recovery are derived from infiltrating monocytes or M1 macrophages^15,19^. So, we examined signal pathways underlying the M2 polarization and found that colony stimulating factor-1 (CSF-1), interferon regulatory factor-4 (IRF4), and peroxisome proliferator-activated receptor gamma coactivator-1α (PGC-1α) expression was significantly decreased in aged kidneys, suggesting impaired M1-M2 conversion during recovery phase of IRI with aging. However, STAT6 and IL-1 receptor-associated kinase-M (IRAK-M) signaling, which are also known factors driving M2 polarization after IRI, were not different between young and aged mice (Fig. 3B).

### *In vitro* polarization into M2 macrophages is not impaired in aged mice

**Figure 4:**
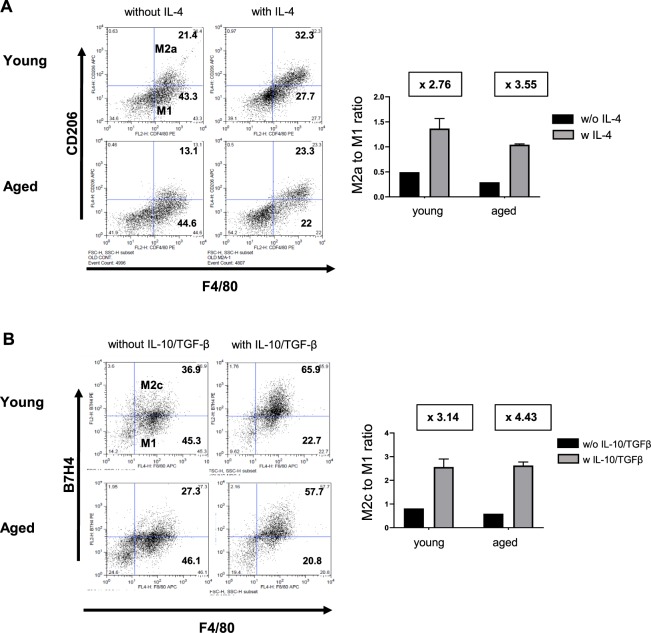
Cytokine-induced M2 polarization of young and aged bone marrow derived mononuclear cells. The differentiation of aged bone marrow (BM)-derived mononuclear cells into (**A**) F4/80^+^ CD206^+^ M2a or (**B**) F4/80^+^ B7H4^+^ M2c macrophages after treatment with IL-4 or IL-10/TGF-b, was not impaired compared to that of young BM derived cell.

We cultured bone marrow derived mononuclear cells from young and aged mice and compared the *in vitro* polarization into M2 macrophages by cytokine stimulation. M2a/M2c polarization was induced by IL-4/IL-13 and IL-10/TGF-β, respectively. The ratio of M2a/M1 and M2c/M1 was determined by flow cytometry (M2a: F4/80 + CD206 + cells, M2c: F4/80 + B7H4 + cells, respectively). Unlike the *in vivo* results, both M2a and M2c polarization were not impaired in aged mononuclear cells, compared to those from young mice (Fig. 4). These results suggest that changes in the intrarenal microenvironment in aged mice after IRI, rather than aging in bone marrow derived monocytes, is more important in impaired M2 polarization after IRI in aged mice. The phagocytic activities of bone marrow derived mononuclear cells isolated from young and aged mice were also compared. There was no significant difference in the percentage of FITC-positive phagocytic cells between the two groups when incubated with FITC-dextran for two hours (Supplementary Fig. 1).

### The number of tubular cells with G1 arrest is significantly higher in aged mice during recovery phase

**Figure 5:**
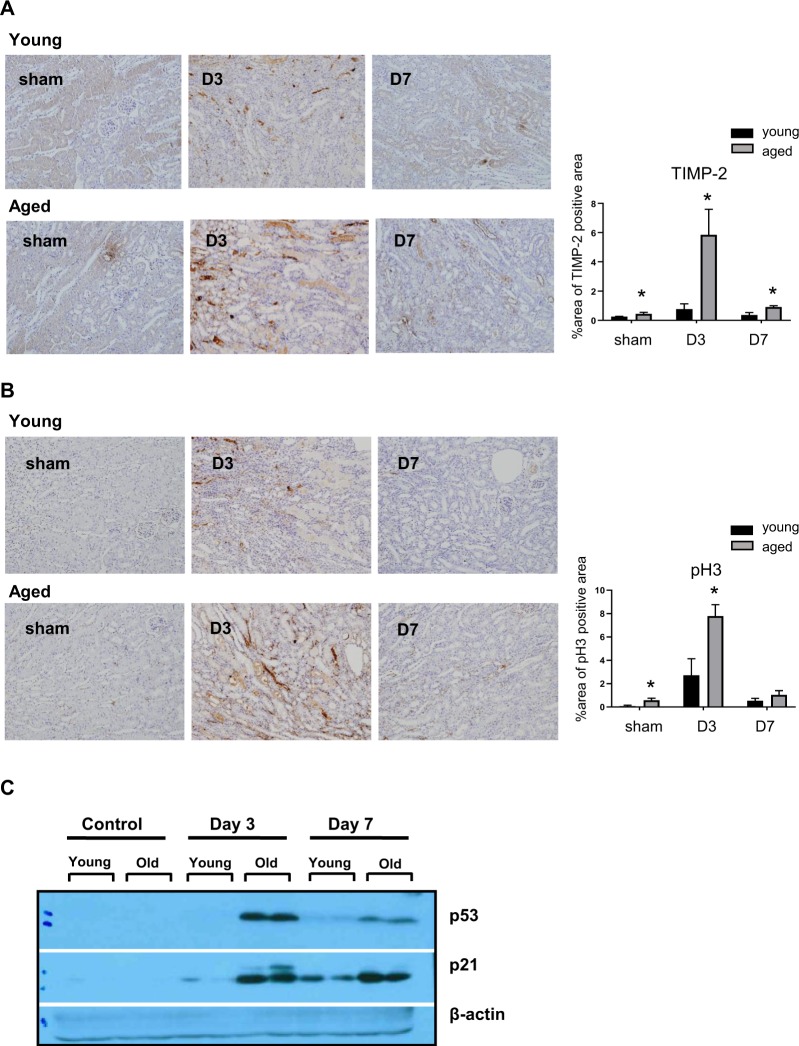
Tubular cell arrest at G1 or G2 phase during recovery phase of young and aged mice. In immunohistochemistry, tubular cells showed significantly elevated (**A**) tissue inhibitor of metalloproteinase-2 (TIMP-2) and (**B**) phospho-Histone H3 (pH3) levels during the recovery phase, along with (**C**) increased p53 and p21 levels in aged mice. Cropped gels are used in the figure, and the full-size gels are presented in Supplementary Fig. S2. Magnification: ×100, *p < 0.05 compared to young mice, n = 4–6 per group.

Since growth arrest is an important phenotype of cellular senescence and might be involved in the alteration of post-IRI microenvironments in aged kidneys, we compared the degree of cell cycle arrest after IRI. Immunohistochemistry showed significantly elevated tissue inhibitor of metalloproteinase-2 (TIMP-2) and phospho-Histone H3 (pH3) levels throughout the recovery phase, along with increased p53 and p21 levels (Fig. 5). Increased expression of G1 cell cycle arrest marker, TIMP-2 lasted longer than that of pH3, a marker for G2-M and these results suggest that G1 cell cycle arrest can be more important phenotypes for altered injury response in aged mice.

### Arrested tubular cells at G1 phase are partially involved in impaired M2 conversion in aged mice

**Figure 6:**
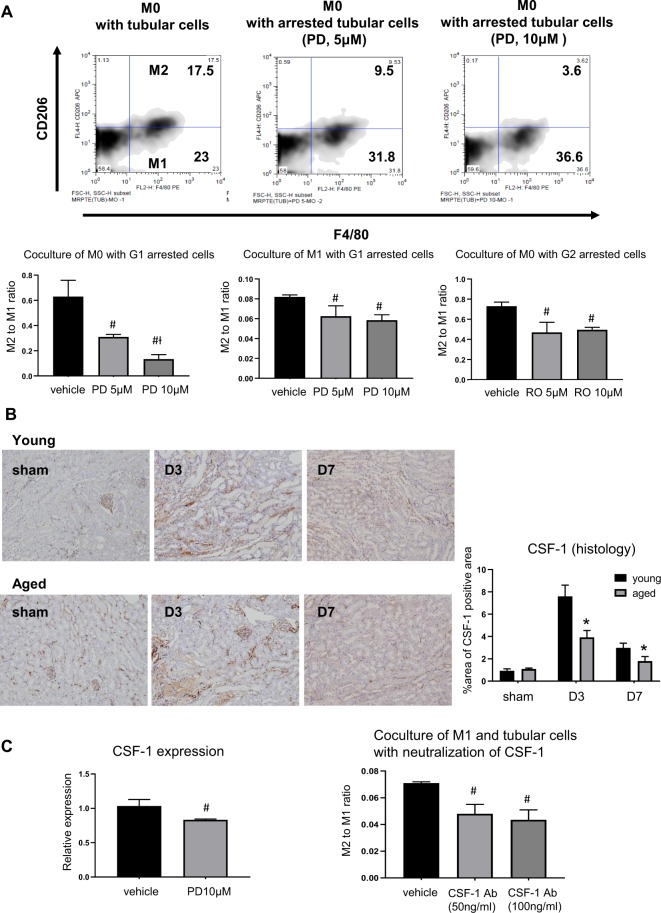
Arrested tubular cells at G1 phase and impaired M2 conversion of aged monocytes and M1 macrophages. Bone marrow derived monocytes or M1 macrophages co-cultured with mouse tubular cells for 72 h using a transwell assay, were converted to M2 macrophages, but (**A**) when monocytes were co-cultured with tubular cells pretreated with PD0332991 (PD), a selective cyclin dependent kinase 4/6 inhibitor, M2 conversion was much decreased dependent on the dose of PD0332991, and when monocytes were co-cultured with tubular cells pretreated with RO3306 (RO), a selective cyclin‐dependent kinase 1 inhibitor, M2 conversion was decreased but not in a dose-dependent manner. M2 conversion from M1 macrophage was also significantly decreased when co-cultured with tubular cells pretreated with PD0332991 (n = 3 per group). (**B**) In immunohistochemistry, tubular cells in aged mice showed low CSF-1 expression, (n = 3–5 per group). (**C**) Mouse tubular cells treated with PD0332991 (PD) showed low mRNA expression of CSF-1, and neutralization of CSF-1 during co-culture of M1 macrophages and mouse tubular cells impaired M2 polarization (n = 3 per group). Magnification: ×100, *p < 0.05 compared to young mice, ^#^p < 0.05 compared to vehicle, ^Ɨ^p < 0.05 compared to PD 5 µM.

To determine the interaction between cell cycle arrest of tubule cells and impaired M2 polarization, we performed in *in vitro* studies using transwell co-culture of mouse tubular cells with bone marrow derived mononuclear cells or M1 macrophages. For induction of G1 or G2 cell cycle arrest, mouse tubular cells were pretreated with PD0332991, a selective cyclin dependent kinase 4/6 inhibitor (Sigma-Aldrich, St. Louis, MO, USA) or RO3306, a selective cyclin‐dependent kinase 1 inhibitor (Sigma-Aldrich) and then were co-cultured with mononuclear cells or M1 macrophages for 72 hours. Mononuclear cells co-cultured with mouse tubular cells showed that 43% of F4/80 + cells were also positive for CD206. In contrast, the percentage of CD206 + M2 macrophage decreased stepwise as the concentration of PD0332991 increased (43%, 23.5%, 9.0% in 0, 5, and 10 µM of PD0332991, respectively). M2 conversion also decreased upon co-culture with RO3306-pretreated arrested cells, but not in a dose-dependent manner. M1 macrophages co-cultured with G1 arrested tubular cells also showed decreased M2 polarization. Taken together, they suggest that G1 cell cycle arrest of tubular cells with senescent phenotypes contributes to the impairment of M2 polarization from both M1 macrophage and monocytes (Fig. 6A).

We also observed that arrested tubular cells had low CSF-1 expression *in vivo and in vitro*. And neutralization of CSF-1 during co-culture of tubular cells and M1 macrophages significantly reduced M2 polarization. These results suggest that the decreased CSF-1/IRF4 signal in arrested tubular cells is partially responsible for the impaired M2 polarization during the recovery phase of IRI in aged mice (Fig. 6B,C).

### Persistent M1-predominant inflammation is associated with progressive fibrosis after ischemia-reperfusion injury

**Figure 7:**
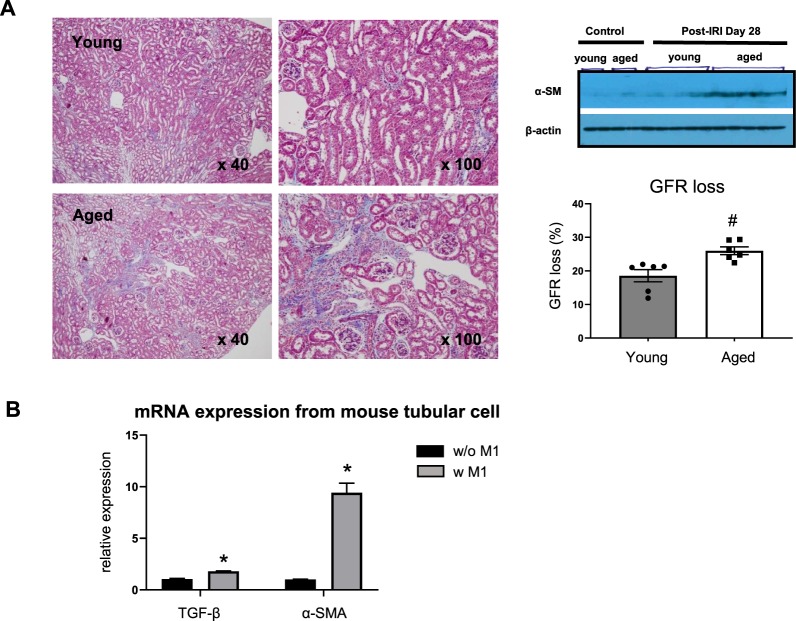
Persistent M1 predominant inflammation and progression to chronic kidney disease. (**A**) In addition to increased M1 related inflammation of aged mice, higher α-smooth muscle actin, severe renal fibrosis and lower glomerular filtration rate (GFR) were observed on day 28 post-IRI in aged mice compared to young mice, Cropped gels are used in the figure, and the full-size gels are presented in Supplementary Fig. S3, GFR loss was calculated as follows: 100 x (GFR difference between pre-IRI and day28 post-IRI) / (pre-IRI GFR), (**B**) Higher mRNA expression of TGF-β and α-smooth muscle actin was observed in tubular cells upon co-culture with M1 macrophages. Magnification: × 40, 100, ^#^p < 0.05 compared to young, *p < 0.05 compared to tubular cells without M1, n = 3–6 per group.

Impaired M2 polarization coupled with increased numbers of G1 cycle-arrested tubule cells led to progression of interstitial fibrosis in aged mice 4 weeks after IRI. This was indicated by Masson-trichrome-stained kidney tissue section and increased protein levels of α-smooth muscle actin. GFR measured by transdermal patch also showed significant reduction in aged mice (Fig. 7A). In order to examine the effect of persistent M1 dominant inflammation on renal fibrosis, renal tubular cells were co-cultured with M1 macrophages for 72 hours using the transwell assay. Higher mRNA expression of TGF-β and α-smooth muscle actin was observed in tubular cells upon co-culture with M1 macrophages (Fig. 7B).

## Discussion

In our study, we demonstrated that defective macrophage M2 polarization, regulated in part by maladaptive tubule cells during the recovery phase of IRI, play an important role in the progression of fibrosis and decline of GFR in aged mice.

Despite numerous epidemiological studies showing the link between AKI and progressive CKD/ESRD in elderly populations^5,11,12^, it still remains unclear whether aging directly affects progression after AKI. Frequently noted comorbid conditions in the elderly can overwhelm the direct effect of aging on AKI outcome. However, our study clearly demonstrated significantly worse functional and histological deterioration following AKI in aged mice. Despite comparable levels of initial injury, GFR decline that was longitudinally measured in the same animals, as well as progression of interstitial fibrosis, were significantly higher in aged mice.

Among several potential mechanisms underlying an accelerated AKI to CKD transition, we hypothesized that persistent inflammation plays an important role. This was based on emerging evidence that showed the dynamic changes in the immune system with aging. Inflammaging refers to chronic low grade inflammation that develops with advanced age and is increasingly recognized as playing an important role in age related diseases^13^. In our study, we also noted that proinflammatory cytokine (i.e., TNF-α, IFN-γ, and IL-12) mRNA levels were significantly elevated in sham operated mice, indicating the presence of inflammaging in the kidneys of aged mice. However, on day 1 after IRI, levels of serum creatinine, NGAL and tubular injury score were not different between aged and young mice. Lack of difference in initial severity of IRI was also demonstrated by Jang *et al*., who showed that aging related changes in intrarenal micromilieu had a small effect on short term outcome of AKI. Regarding the transition to CKD, Sato *et al*. demonstrated the important role of tertiary lymphoid tissues (TLTs) by overproduction of proinflammatory cytokines in aged mice. Targeting TLT formation using anti-CD4 monoclonal antibodies showed the potential to ameliorate renal fibrosis and inflammation^20^.

We focused on macrophage polarization during the recovery phase and the development of persistent inflammation. Dynamic macrophage polarization is increasingly recognized as an important player in injury/repair processes in AKI^15,16^. In our study, we observed that the number of CD206 + M2 macrophages and arginase expression were significantly decreased, while iNOS expression was significantly increased in aged kidneys, compared to young kidneys during the recovery phase. Although the origin of M2 macrophages during AKI recovery may be heterogeneous, previous reports have shown that M2 macrophages are derived from infiltrating monocytes or M1 macrophages in the IRI recovery^15,19^. Therefore, these data suggest that defective M2 polarization could be one mechanism leading to persistent chronic inflammation after IRI. Persistent M1 macrophage-mediated inflammation has been demonstrated to lead to tubular atrophy and interstitial fibrosis. According to Lech *et al*., persistent macrophage polarization toward a proinflammatory phenotype in IRAK-M deficient mice led to impaired kidney regeneration and promoted CKD^21^. Zhang *et al*. also noted that deletion of IL-4/IL-13, important Th2 cytokines for M2 polarization, resulted in tubulointerstitial fibrosis with increased M1 and decreased M2 markers^22^. However, unlike these two studies showing the importance of IRAK-M or Th-2 cytokines, defective CSF-1 signaling seems to be responsible for persistent M1-mediated inflammation in aged mice. CSF-1 from tubular cells is known to drive M2 polarization through mammalian targeting of rapamycin complex 2 (mTORC2) activation, leading to the expression of IRF4^23^. We observed that increased CSF-1 and downstream IRF4 expression normally seen in young mice during the recovery phase were significantly blunted in the kidneys of aged mice, suggesting that this might be one of the mechanisms of impaired macrophage M2 polarization in aged mice. PGC-1α and PGC-1β expression regulates M2 polarization via interaction with PPAR-γ^24,25^ and showed a significant decrease in aged kidneys. However, the downstream signal STAT6, as well as IRAK and IL-4/IL-13 expression, were not different between aged and young mice.

In contrast to current data showing persistent inflammation, our group previously demonstrated that young mice who were transplanted with senescent bone marrow (BM) from aged mice demonstrated significantly attenuated kidney inflammation and injury, as compared to aged mice transplanted with BM from young mice^26^. This clear discrepancy in the phenotype of post-ischemic senescent immune cells led us to hypothesize that the intrarenal microenvironment surrounding immune cells might be more important in driving macrophage polarization. As expected, *in vitro* macrophage polarization experiments demonstrated that there was no defect in M2a or M2c polarization by cytokine cocktail stimulation in aged bone marrow derived mononuclear cells, compared to young. These data suggest that it is not a defect intrinsic to senescent macrophages but primarily comes from the altered microenvironment of post-ischemic kidneys of aged mice.

We next examined the possible altered injury response of tubule cells as a culprit for defective macrophage polarization in aged mice. Because cell growth arrest and failure to divide are important phenotypes of cellular senescence, we evaluated cell cycle arrest of tubule cells. Ischemic or nephrotoxic AKI animal models have demonstrated that G2-M arrest of tubule cells with a shift to a profibrotic phenotype via JNK signaling is an important mechanism in the transition from AKI to CKD^27^. In our study, we noted that the number of TIMP-2 expressing tubule cells and protein expression level increased significantly exclusively in aged kidneys. This change was also accompanied by increased p53 and p21 expression, showing that substantial numbers of tubule cells are arrested in the G1 cell cycle. The importance of G1 cell cycle arrest in the progression of fibrosis has been also demonstrated in AKI to CKD models. Lim *et al*. showed that IRI in a remnant kidney model increased G1 cell cycle arrest during the recovery phase and that treatment with a p53 inhibitor decreased the expression of G1 arrest markers, as well as fibrosis^28^. These results suggest that prolonged epithelial G1 cell cycle arrest might be partially responsible for impaired recovery from AKI superimposed on CKD. Despite data showing that epithelial cell cycle arrest promotes fibrosis, no study has been conducted to determine whether cell cycle arrest affects macrophage polarization. Therefore, to clarify the direct effect of arrested tubule cells on macrophage polarization, we performed *in vitro* experiments using a transwell system. Bone marrow derived mononuclear cells or M1 macrophages co-cultured with mouse tubular cells for 72 hours showed increased M2 polarization. However, when mononuclear cells were co-cultured with tubule cells pretreated with an inducer of G1 arrest, PD 0332991, the percentage of M2 macrophages decreased dose dependently. These results show that arrested tubule cells directly affect or program macrophage polarization. In addition, we also found that TGF-β and α -smooth muscle actin expression in arrested tubular cells was highest when these were cultured with macrophages of the M1 phenotype. Renal fibrosis *in vivo* was also increased significantly in aged mice with M1 related inflammation at 28 days following IRI. In aged mice, prolonged G1 cell cycle arrest and persistent low grade M1-predominant inflammation were thought to have contributed to fibrosis by interacting with each other.

In conclusion, we demonstrated that defective macrophage M2 polarization during the recovery phase, partially driven by senescent tubule cells with cycle arrest in the kidney microenvironment, plays an important role in persistent inflammation and the development of tubulointerstitial inflammation in the elderly. In the future, strategies that aim to change aging-induced injury responses, such as inflammaging, are needed to facilitate recovery and may be key to the treatment of elderly patients with AKI and CKD.

## Materials and Methods

### Experimental animals and renal ischemia-reperfusion injury

Six- to eight-week-old male C57BL/6 mice (weight, 20~25 g) were purchased from Orient (Seongnam, Korea). The age of aged mice was up to 48 weeks. All experimental protocols were approved by the animal care committee of Korea University (IRB number: KOREA-2017-0158) and followed the NIH publication “Principles of Laboratory Animal Care.” All mice had free access to water and regular chow. IRI was done on a warm surgical table to maintain the body temperature at 37 °C. To induce IRI, mice were anesthetized with an intraperitoneal (i.p.) injection of 15 mg/kg of ketamine and 2.5 mg/kg of xylazine and were then subjected to bilateral renal pedicle clamping for 25 minutes. After the clamps were removed, reperfusion of kidneys was observed for 1 minute. A sham operation was performed in a similar manner, except without the clamping of renal pedicles.

### Flow cytometric analysis

Flow cytometric analyses of kidney cells were performed using anti-F4/80-PE/APC, anti-CD206-APC, and B7-H4-PE antibodies purchased from BD Biosciences (San Jose, CA, USA) or from eBioscience (San Diego, CA, USA).

### Histological analysis

Tubular injury was assessed using periodic acid-Schiff (PAS)-stained kidney sections. For immunohistochemical staining, we used rat anti-mouse F4/80 (Serotec, Kidlington, UK), Gr-1 (eBioscience, San Diego, CA, USA), CSF-1 (Abcam, Cambridge, UK), TIMP-2 (Abcam), pH3 (Abcam) antibodies. A total of 8–10 high power fields (HPFs) were captured, and the mean number of positive cells was compared between groups.

### Real-time polymerase chain reaction

Real-time RT-PCR was performed using the iCycler IQ real-time PCR detection system (Bio-Rad, Hercules, CA, USA) to detect TNF-α, iNOS, IFN-γ, IL-12, IL-10, TGF-β, α -smooth muscle actin and arginase-1 expression levels in the kidney. The reference gene used was 18 s rRNA (RT2 PCR Primer Set, Applied Biosystems, Foster City, CA, USA).

### Transdermal glomerular filtration rate measurement

Glomerular filtration rate (GFR) was measured noninvasively by recording the transcutaneous fluorescence of FITC-sinistrin over time by attaching a fluorometer device (Medi-Beacon, St. Louis, MO, USA) to the mice. The fluorometer was affixed to the shaved back of anesthetized mice using a double-sided adhesive patch; the background fluorescence level of the skin was recorded for 1 minute, and FITC-sinistrin (15 mg/kg; Fresenius-Kabi, Linz, Austria) was then injected intravenously via the retro-orbital sinus. The fluorometer was programmed to make a transcutaneous measurement every 5 seconds; measurements were made for 5 hours and stored on the device. GFR was calculated using a single-compartment model, enabling direct conversion from the elimination half-life, using a published conversion factor.

### *In vitro* study

Bone marrow cells were isolated from the femur and tibia of 8- to 10-week-old C57BL/6 mice. Cells were differentiated into bone marrow-derived macrophages in RPMI-1640 culture medium with 10% heat-inactivated fetal bovine serum (FBS) and 1% penicillin streptomycin (all Gibco Invitrogen, Breda, The Netherlands) for 8 days. Differentiated macrophages were harvested and re-cultured for 48 hours with normal medium to form M0 macrophages, with IFN-γ (100 U/ml, Hycult Biotech, Uden, Netherlands) to form M1 macrophages, with IL-4 (20 ng/ml, R&D Systems, Minneapolis, MN, USA) to form M2a macrophages, and with IL-10/TGF-β (each 10 ng/ml, R&D Systems) to form M2c macrophages. For the transwell co-culture system, mouse proximal tubular cells were cultured in a monolayer on a transwell membrane and were incubated with bone marrow-derived mononuclear cells or M1 macrophages with or without mouse M-CSF antibody (50 or 100 ng/ml, R&D Systems, Minneapolis, MN, USA) for 72 hours. To confirm the polarization of macrophages, they were analyzed using a flow cytometer after staining with F4/80, B7-H4, and CD206 antibodies, respectively.

## Supplementary information

Supplementary figures
