# Supplementary material for: Impact of aging on transition of acute kidney injury to chronic kidney disease
Source: Sci Rep. 2019 Dec 5;9:18445. doi: 10.1038/s41598-019-54585-1 (PMC6895109; doi:10.1038/s41598-019-54585-1)
Supplement: Supplementary file 1 — Supplementary figures [file 41598_2019_54585_MOESM1_ESM.pdf]

Impact of aging on transition of acute kidney injury to chronic kidney disease

Myung-Gyu Kim[1], Jihyun Yang[1], Yoon Sook Ko[1], Hee Young Lee[1], Se Won Oh[1], Won Yong Cho[1], Sang-Kyung Jo[1]*

1. Department of Internal Medicine, Korea University Medical College, Seoul, Korea

Corresponding Author:

Sang Kyung Jo, MD. PhD, Professor

Department of Internal Medicine, Korea University Medical College

Address: Korea University Anam Hospital, 5Ka, Anam-Dong, Sungbuk-Gu, Seoul, Korea

Tel: 82-2-920-5909

Fax: 82-2-927-5344

E-mail: sang-kyung@korea.ac.kr

Supplementary Figure S1

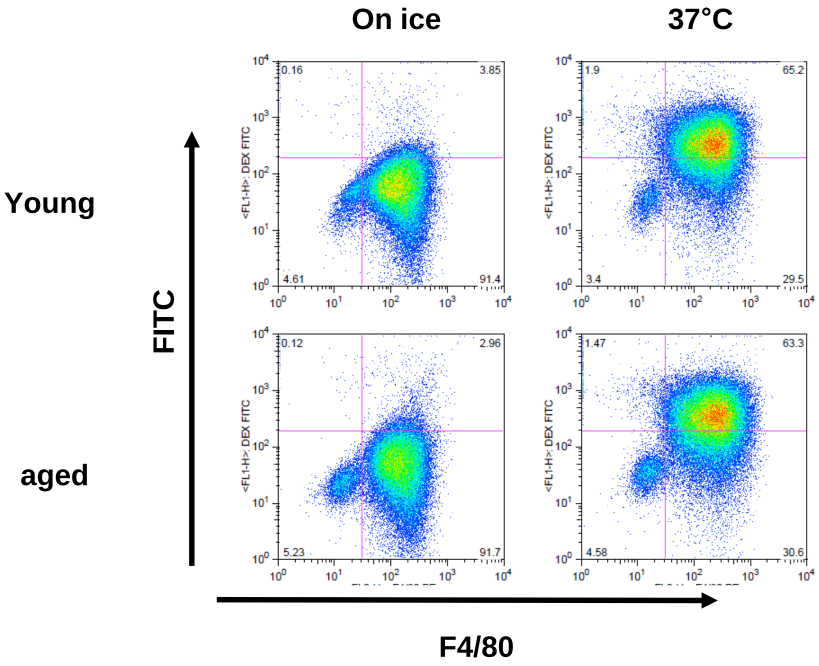

Supplementary Figure S1. The phagocytic activities of bone marrow (BM) derived mononuclear cells isolated from young and aged mice. BM derived monocytes were incubated with FITC-conjugated dextran for 48 hours at 37˚C or 4˚C and percentage of FITC-positive phagocytic cells between the two groups were compared.

Supplementary Figure S2

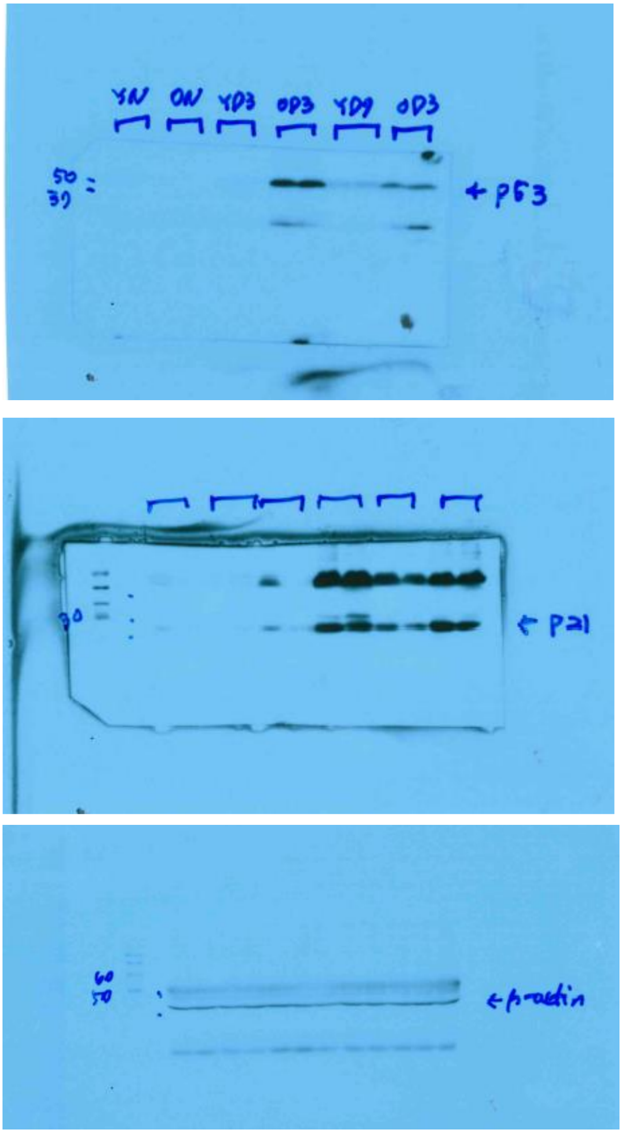

Supplementary Figure S2. Full length blots of Figure 5C. Brightness was adjusted during processing these gels

Supplementary Figure S3

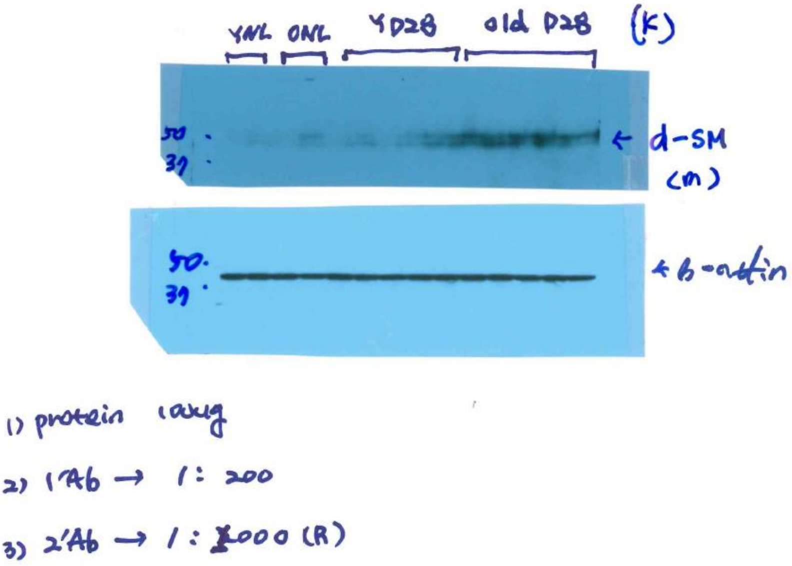

Supplementary Figure S3. Full length blots of Figure 7A. Brightness was adjusted during processing these gels
